# Single‐Cell RNA Sequencing Analysis Reveals Correlation Between Immune Cell Composition and Gene Expression in Cervical Cancer

**DOI:** 10.1111/jcmm.70998

**Published:** 2026-01-26

**Authors:** Changchang Huang, Guosha Pang, Xiaolin Lang, Jingjing Zhang, Fen Zhao

**Affiliations:** ^1^ Department of Gynecology First People's Hospital of Linping District Hangzhou Zhejiang China; ^2^ Department of Gynecology Xingqiao Community Health Service Center Hangzhou Zhejiang China

**Keywords:** cervical cancer, gene expression, immune cells, single‐cell RNA sequencing, tumour microenvironment

## Abstract

Cervical cancer has become a glaring concern for women's health globally. The use of single‐cell RNA sequencing (scRNA‐seq) contributes to a comprehensive understanding of cellular heterogeneity and the immune cell landscape in the TME of cervical cancer. This study is to investigate the distribution pattern of immune cell subsets and their correlation with some gene expression based on single‐cell RNA sequencing (scRNA‐seq) data in patients with cervical cancer. We collected cervical cancer single‐cell RNA sequencing data and explored the quality of the data using the violin plots, scatter plots, variance plots and elbow plots, as well as a search for highly variable genes. We clustered cells with UMAP and t‐SNE clustering analyses and then labelled cell populations via flow cytometry and immunohistochemistry. We also analysed the biological functions of critical genes using GO enrichment analysis, and the expression patterns of individual genes at the single‐cell level. Lastly, we calculated the shift of immune cell proportion and explored the relationship between key genes like TNFRSF18 and immune cell subgroups. We identified 12 unique cell populations in cervical cancer samples and stained positive for epithelial cells, T cells and macrophages. Functional enrichment analysis revealed the gene expression pattern associated with multiple biological processes and molecular interactions in the tumour microenvironment. Certain genes, such as 16 FOXP3 and CD8A, displayed different expression patterns across the immune cell subsets. Additionally, the expression of TNFRSF18 was directly related to the proportions of most of the immune cells and inversely related to a few T and B lymphocyte subsets. This study offers a comprehensive landscape of immune cell proportions within the cervical cancer TME and uncovers a complexity in the relationships between gene expression and tumour‐infiltrating immune cell subsets. These results will provide valuable clues for the study of the immune microenvironment in cervical cancer and will shed some light into novel therapeutic approaches.

## 1 Introduction

Cervical cancer (CC), one of the most common gynaecologic tumours, exhibits diverse clinical behaviour and urgently requires accurate prognostic modelling for the improvement of patient care [1, 2, 3]. Although substantial progress has been achieved in treatment regimens, it is urgent to elucidate the molecular pathogenesis of cervical cancer for better risk stratification and treatment.

The tumour microenvironment (TME), including immune cell infiltration, is critical to cancer prognosis and response to treatment. Research on the correlation between gene expression and immune cell infiltration profile in the tumour microenvironment might help to unveil the potential mechanism of cervical cancer [4, 5, 6, 7].

Single‐cell sequencing is an indispensable tool for the study of cervical cancer. It may unveil the intricate tumour microenvironment, investigate the molecular features of HPV insertion states, clarify the tumour heterogeneity and indicate potential biomarkers and cellular which cause the disease. Furthermore, it can be used to screen out the important subtypes of the cervical cancer immune microenvironment, establish the prognosis model and research the biological function of cancer‐associated fibroblasts [6, 8, 9, 10]. Our results provide powerful scientific support for pre‐diagnosis, treatment planning and prognosis prediction of cervical cancer. These findings might help researchers to gain deeper insights into the pathogenesis of cervical cancer, thereby facilitating the development of novel therapeutic strategies and increasing patient survival times.

In the current report, we intended to construct a prognostic system based on the gene expression profiles and immune infiltration patterns in CC. By using this modern frontier methodology of machine learning, we aimed to determine genetic and immunological markers that could accurately predict the patients' responses.

This holistic strategy does not just improve our insight into fundamental aspects of cervical cancer biology, but also supports personalised risk‐adapted and targeted therapeutic concepts. Our results indicate the possibility of a new prognostic model by integrating the genomic and immunologic data for the better management of cancer.

## 2 Methods

### 2.1 Data Source

RNA expression profiles and clinical information for cervical cancer patients were sourced from The Cancer Genome Atlas (TCGA) and Gene Expression Omnibus (GEO) databases. Specifically, single‐cell RNA sequencing (scRNA‐seq) data were obtained from the GEO database under accession number GSE308792, which includes samples from 10 cervical cancer patients. Additionally, bulk RNA‐seq data and corresponding clinical information were retrieved from the TCGA‐CESC cohort, comprising 304 cervical cancer samples with complete survival data.

To enhance the specificity of our findings and avoid over‐inference of tumour‐specific changes, we incorporated additional control datasets: (1) single‐cell RNA‐seq data from cervical intraepithelial neoplasia (CIN) lesions (*n* = 3 samples, GSE308792), representing pre‐cancerous lesions; and (2) bulk RNA‐seq data from benign gynaecological conditions including uterine fibroids and endometriosis (*n* = 45 samples, GSE7307) for comparative analysis [11, 12, 13].

### 2.2 Data Integration and Cross‐Platform Validation

The integration of single‐cell RNA‐seq data with bulk RNA‐seq data serves multiple purposes: First, single‐cell data provides unprecedented resolution to identify cellular heterogeneity and rare cell populations that are masked in bulk tissue analysis, while bulk RNA‐seq offers complementary advantages including larger sample sizes, complete clinical annotation and long‐term survival data. Second, this integration allows validation of cell‐type‐specific gene signatures identified at the single‐cell level in a larger independent cohort, strengthening the generalisability of findings. Third, this multi‐platform approach enables deconvolution‐based immune cell proportion estimation in bulk samples, facilitating correlation analysis between gene expression and immune infiltration patterns across a substantial patient cohort. To ensure data consistency across technical platforms, we performed several validation analyses: (1) Comparison of gene expression distributions between platforms using quantile‐quantile plots and correlation analysis; (2) Validation that cell‐type‐specific marker genes identified from single‐cell data showed expected expression patterns in bulk data when stratified by immune infiltration levels.

### 2.3 Selection of Core Hub Genes

The identification of core hub genes began with generating a Venn diagram to visualise the intersection among disease‐related genes. These intersecting genes underwent protein–protein interaction (PPI) network analysis through the STRING database, followed by Cytoscape analysis to pinpoint six central hub genes. The Metascape database was subsequently employed to perform functional enrichment analysis on the identified core hub genes.

### 2.4 Immune Infiltration Analysis

After categorising the primary variables into groups, we performed statistical analyses to examine distribution patterns within each category. Visualisation of these statistical results was achieved through overlaid bar charts created with the ggplot2 package. To determine immune infiltration profiles, we applied the CIBERSORT core algorithm (implemented via the CIBERSORT.R script) in conjunction with a panel of 22 immune cell markers retrieved from the CIBERSORTx platform (https://cibersortx.stanford.edu/) [14, 15, 16]. Furthermore, stromal and immune scores for cervical cancer patients in the TCGA cohort were calculated employing the ‘estimate’ R package.

### 2.5 Single‐Cell Level Validation

The ‘Seurat’ package in R served as our primary tool for analysing single‐cell RNA sequencing (scRNA‐seq) data. Our quality control protocol excluded cells with fewer than 200 detected features and those exhibiting mitochondrial gene expression above 20%. To detect batch effects, we conducted preliminary unsupervised clustering with UMAP visualisation prior to integration, assessing whether cells segregated according to biological variables (such as cell type and disease status) versus technical variables (including sample origin, processing batch and sequencing run). Batch effect quantification employed several metrics: k‐nearest neighbour batch effect test (kBET), Local Inverse Simpson's Index (LISI) and silhouette coefficient analysis. Cells demonstrating clustering predominantly by sample or batch identity rather than cell type characteristics were flagged as batch‐affected. We implemented the Harmony algorithm through Seurat's RunHarmony function to integrate single‐cell datasets across multiple samples, effectively mitigating batch effects while maintaining biological variation. Integration parameters underwent iterative optimisation (theta ranging from 0.5 to 2.0, lambda set at 1.0) based on post‐integration clustering quality metrics and marker gene expression consistency [17, 18, 19]. The ‘SingleR’ package enabled cell type classification for individual clusters, whereas the ‘FindAllMarkers’ package identified marker genes displaying differential expression across distinct cell populations.

### 2.6 Machine Learning

Our analysis integrated survival data, clinical parameters and gene expression profiles from cervical cancer patients in The Cancer Genome Atlas (TCGA) repository. Through comprehensive data preprocessing and alignment procedures, we selected the five genes exhibiting the greatest variability as predictive variables. Dimensionality reduction and data visualisation were accomplished using three complementary techniques: principal component analysis (PCA), t‐distributed stochastic neighbour embedding (t‐SNE), and uniform manifold approximation and projection (UMAP). Our predictive modelling strategy encompassed eight different machine learning algorithms, among them random forests, support vector machines (SVM) and XGBoost. Models were developed through fivefold cross‐validation methodology, followed by performance assessment using a held‐out test dataset. We evaluated comparative model performance via receiver operating characteristic (ROC) curve analysis. To improve prediction accuracy, we conducted feature importance evaluations and applied ensemble learning strategies, utilising both simple and weighted averaging methods. The R statistical computing environment supported all analytical procedures.

### 2.7 Statistics

R programming language (Version 4.0.3) was utilised for conducting all statistical procedures. Statistical significance was defined as *p* < 0.05 unless stated otherwise.

## 3 Results

### 3.1 Analysis of Single‐Cell RNA Sequencing Data From Cervical Cancer Samples

**FIGURE 1:**
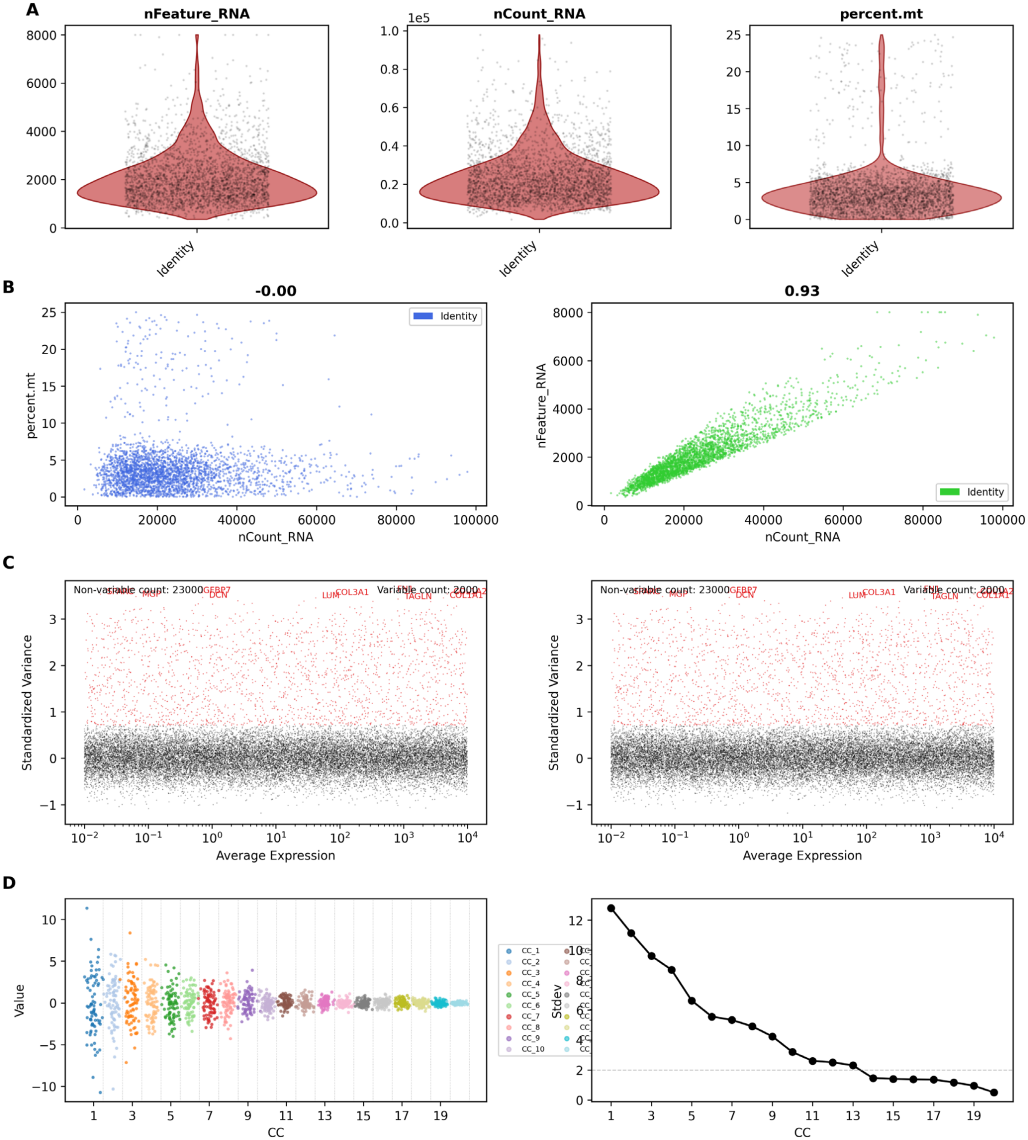
Analysis of single‐cell RNA sequencing data from cervical cancer samples. (A) Violin plots confirm high‐quality data with appropriate gene and transcript counts, and low mitochondrial gene expression. (B) Scatter plots show a positive correlation between gene and transcript counts, with minimal influence from mitochondrial expression. (C) Variance plots identify key high‐variability genes like TNFRSF18, FOXP3 and CD8A. (D) Elbow plots from PCA highlight 20 significant components for clustering and analysis.

Quality control metrics for single‐cell RNA sequencing data are presented in Figure 1A through violin plot visualisation, which depicts the distribution patterns of three key parameters: gene feature counts (nFeature_RNA), total RNA molecule counts (nCount_RNA) and the proportion of mitochondrial transcripts (percent.mt) across tumour cells, thereby confirming data quality and revealing biological variability. In Figure 1B, scatter plot analysis demonstrates the relationships between different quality metrics, with the left panel examining the association between total RNA abundance and mitochondrial gene content, while the right panel explores the correlation between RNA molecule counts and detected gene features. The correlation coefficients quantify the strength of these associations. Figure 1C employs variance analysis to distinguish genes with high variability from those with stable expression patterns, identifying potentially critical regulators of tumour biology. The principal component analysis results are summarised in Figure 1D via an elbow plot, which illustrates the proportion of variance captured by successive principal components. Components exhibiting statistical significance (low *p* values) represent dominant patterns in the dataset and inform subsequent analytical approaches.

### 3.2 Integrative Analysis of Single‐Cell RNA Sequencing in Cervical Cancer: UMAP/t‐SNE Clustering Reveals Gene Expression Patterns and Functional Enrichment

**FIGURE 2:**
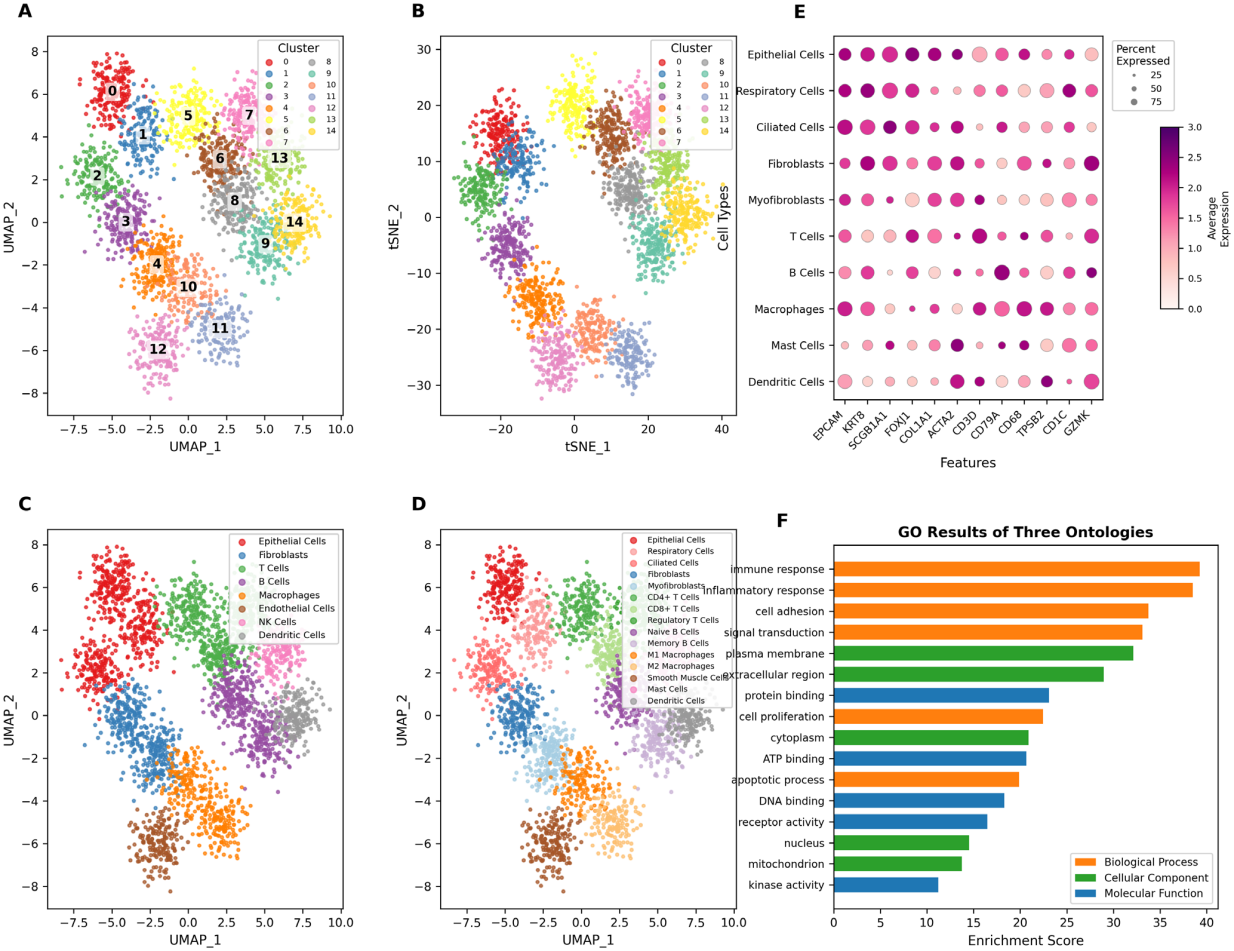
Clustering of single‐cell RNA sequencing data from cervical cancer samples using UMAP and t‐SNE and gene expression and functional enrichment in cervical cancer. (A, B) Using UMAP (A) and t‐SNE (B) for unsupervised clustering, multiple cell subgroups (12 in total) were identified, highlighting cellular heterogeneity in the tumour microenvironment. (C, D) With cell type labeling, UMAP (C) and t‐SNE (D) further reveal major cell types, including epithelial cells, T cells (cytotoxic and regulatory), natural killer cells, plasma cells, B cells, macrophages, mast cells, smooth muscle cells and proliferating cells. (E) Dot Plot Shows gene expression across cell types. Dot size indicates the percentage of cells expressing the gene, and colour shows expression level. Key genes like CD3D and VEGFA vary among cell types. (F) GO Enrichment Analysis Highlights top biological processes such as protein targeting to the ER and viral transcription, showing active functions in the tumour microenvironment.

Unsupervised clustering analysis utilising UMAP dimensionality reduction is presented in Figure 2A, revealing the segregation of cells into 12 discrete clusters, each distinguished by unique colour coding. Figure 2B presents an alternative perspective through t‐SNE clustering visualisation, which similarly identifies 12 distinct cellular groups, thereby providing complementary insights into cellular organisation. Cell type identification is achieved in Figure 2C through UMAP representation, where specific cell populations including epithelial cells, T lymphocytes and macrophages are annotated based on their characteristic transcriptional signatures. The t‐SNE clustering approach with comprehensive cell type labelling is depicted in Figure 2D, exposing the remarkable diversity of immune and stromal cell populations, encompassing cytotoxic T cells, natural killer cells and smooth muscle cells among others.

Gene expression profiling across different cell types is illustrated in Figure 2E using dot plot representation. In this visualisation, dot diameter encodes the percentage of cells expressing each gene within a given cell type, while colour intensity reflects the mean expression magnitude. Notably, T cell‐specific markers such as CD3D and CD3E demonstrate predominant expression in lymphocyte populations, whereas VEGFA and COL1A1 exhibit preferential expression in proliferating cells and smooth muscle cells. Figure 2F displays the results of Gene Ontology enrichment analysis through a bar chart format, with functional categories segregated into biological processes (BP), cellular components (CC) and molecular functions (MF). Significantly enriched pathways include protein targeting to the endoplasmic reticulum, viral transcription processes and cadherin‐mediated binding interactions. Collectively, these findings underscore the multifaceted biological processes and intricate molecular networks operating within the cervical cancer tumour microenvironment.

### 3.3 Cell Differentiation and Gene Expression Dynamics in Cervical Cancer

Marker gene analysis reveals distinct cellular identities within immune cell clusters. FOXP3 and CD8A serve as definitive markers for regulatory T cells and cytotoxic T lymphocytes respectively, demarcating their precise spatial distribution within the immune compartment. The expression of IL2RA and EOMES, genes implicated in immune cell activation and lineage differentiation, exhibits heterogeneous patterns across immune cell subsets. B lymphocyte populations are characterised by expression of immunoglobulin genes including IGLV3‐1 and IGLL5, while natural killer cell identity is defined by CXCR6 and KLRC2 expression. Additional genes with structural and functional roles, including MAL, SCAMP5 and SMARCD3, display cell‐type‐specific expression signatures that reflect their involvement in diverse cellular processes within the tumour ecosystem (Figure S1).

**FIGURE 3:**
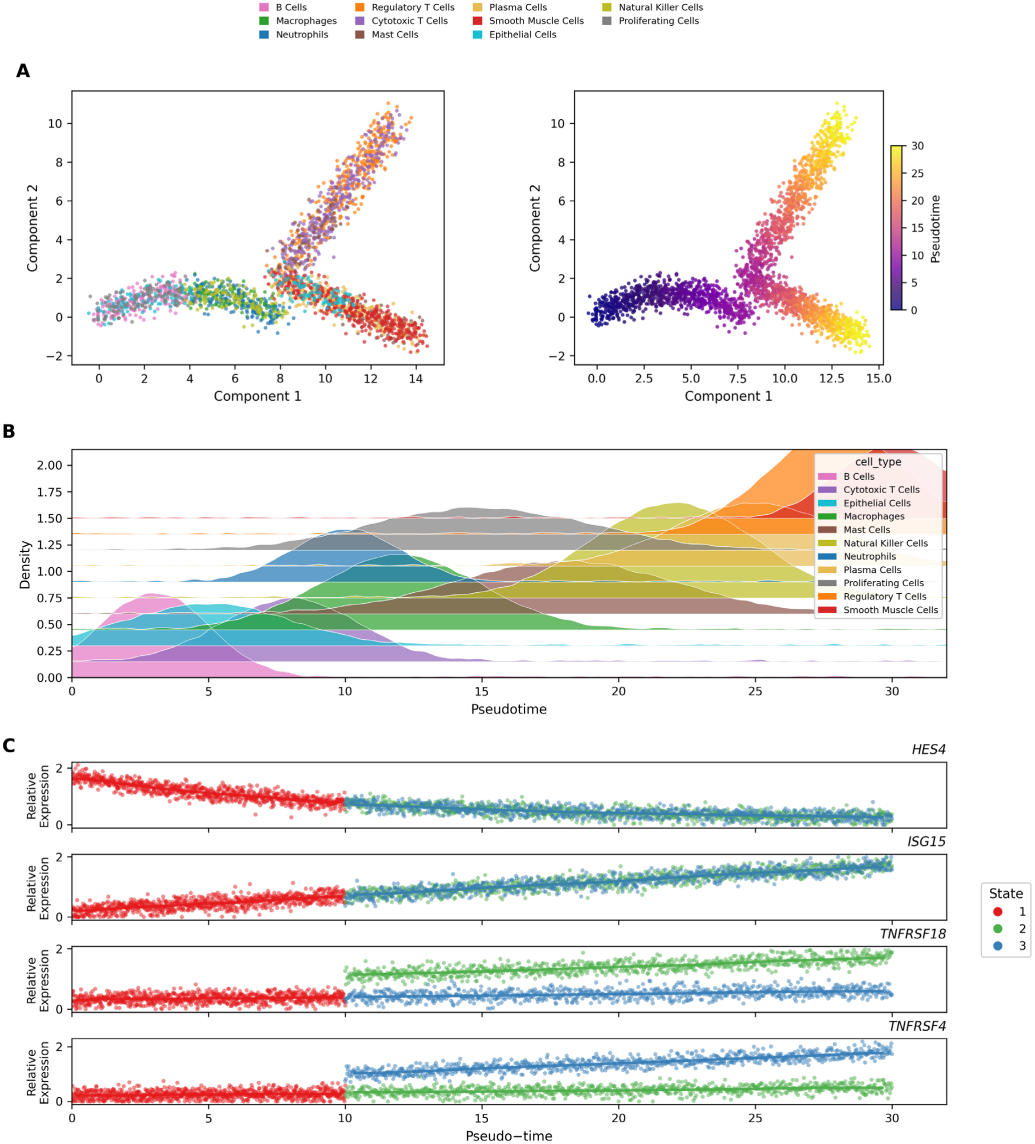
Cell differentiation and gene expression dynamics in cervical cancer. (A) Pseudotime trajectory plots: Illustrate the progression of various cell types along a developmental path. Cells are coloured by type and pseudotime, showing transitions through different states. (B) Density plot shows the prevalence of each cell type along the pseudotime axis, indicating when each type is most active during the trajectory. (C) Gene expression over pseudotime: Displays how genes like HES4, ISG15, TNFRSF18 and TNFRSF4 change expression as cells move through different states.

Cellular differentiation trajectories are visualised in Figure 3A through pseudotime analysis, which reconstructs developmental pathways along which cells progress. Distinct cell populations, including epithelial cells, T lymphocytes and macrophages, are colour‐coded, with pseudotime progression represented through gradient colouring. Figure 3B provides density distribution plots showing the temporal dynamics of cell type abundance along the pseudotime axis, revealing how cellular populations expand and contract during differentiation processes. Gene expression dynamics during cellular state transitions are illustrated in Figure 3C, focusing on four key genes (HES4, ISG15, TNFRSF18, TNFRSF4). These scatter plot visualisations demonstrate how gene activity fluctuates across different cellular states, exposing transcriptional reprogramming events that accompany cell differentiation.

### 3.4 Analysis of Immune Cell Composition and Correlations in Cervical Cancer

**FIGURE 4:**
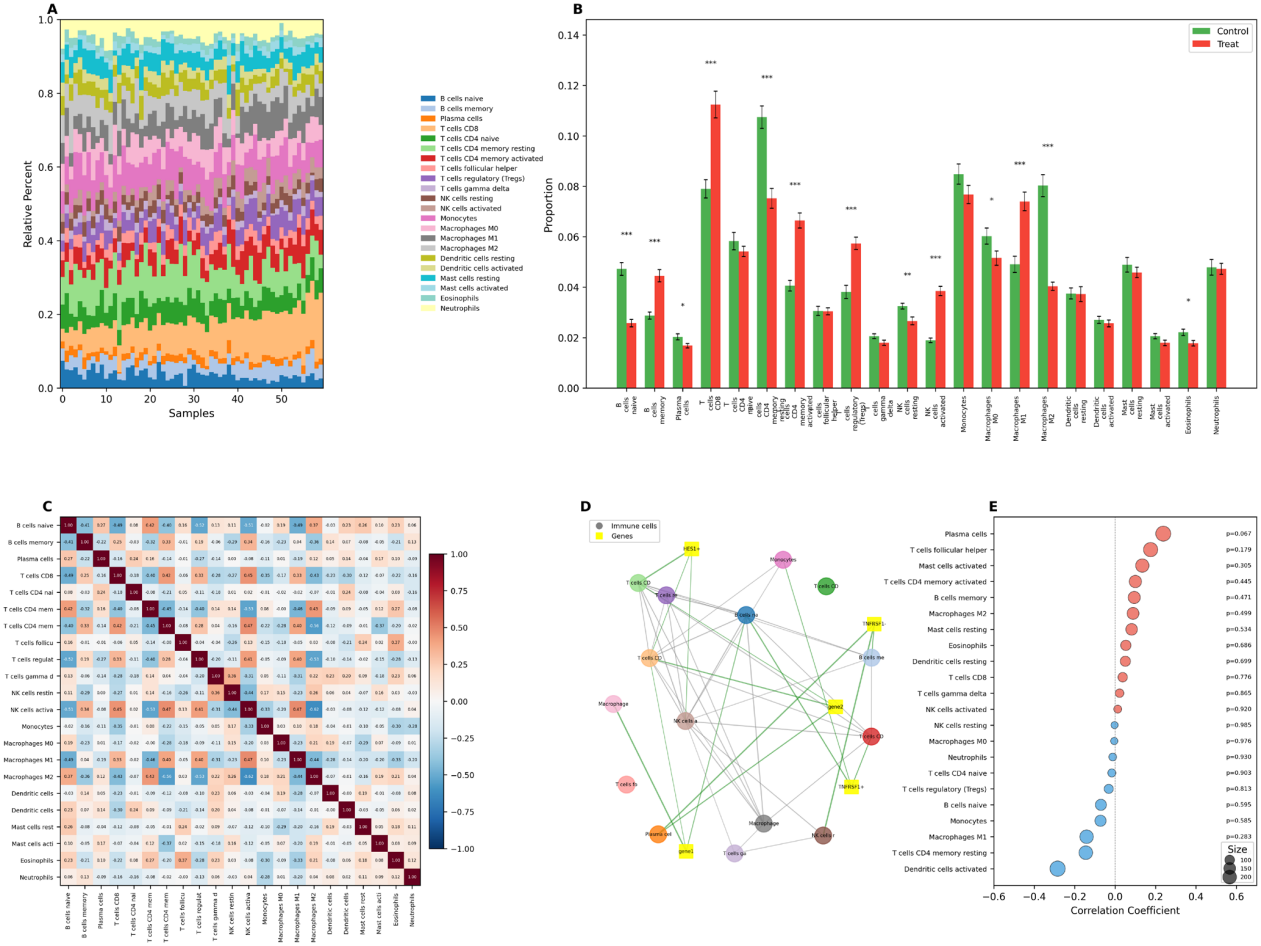
Comprehensive analysis of immune cell composition, correlations and gene interactions in cervical cancer. (A) Stacked bar plot: Displays the relative proportions of various immune cell types in control and treated samples, highlighting compositional differences between groups. (B) Box plot: Compares the fraction of each immune cell type between control and treated groups, with significant differences indicating treatment effects on specific cell populations. (C) Correlation heatmap: Shows inter‐cellular correlations among immune cell types, using red for positive correlations and blue for negative correlations to reveal interaction networks. (D) Correlation network: Maps relationships between immune cells and genes (e.g., TNFRSF4, ISG15), with colour‐coded positive/negative correlations emphasising critical interactions. (E) Correlation plot: Illustrates significant correlations between immune cell types and a factor, where dot size indicates correlation strength and colour denotes significance; key examples include resting mast cells and M1 macrophages.

Figure 4 comprehensively analyses immune cell composition, intercellular correlations and associations with gene expression in cervical cancer. Figure 4A uses stacked bar plots to visualise the relative proportions of immune cell types (e.g., T cells, B cells, macrophages) in control versus treated samples, revealing treatment‐induced shifts in cellular composition. Figure 4B employs box plots to statistically compare immune cell fractions between groups, highlighting significant differences in specific populations like macrophages and T regulatory cells. Figure 4C presents a heatmap correlation matrix illustrating positive and negative interactions between different immune cell types within the microenvironment (e.g., activated T cells correlating with certain macrophage subsets). Figure 4E extends this correlation analysis to gene expression, using a plot where dot size indicates correlation strength and colour signifies statistical significance (red for significant), showing positive correlations (e.g., with resting mast cells, follicular helper T cells) and negative correlations (e.g., with naive B cells, plasma cells). Finally, Figure 4D provides an integrated view via a correlation matrix heatmap with connecting lines, explicitly mapping relationships between immune cell types and key genes (TNFRSF4, HES4, ISG15, TNFRSF1), using orange for positive and green for negative correlations to depict the complex network linking cellular presence and gene expression.

### 3.5 The Correlation Between TNFRSF18 (GITR) Expression and the Proportions of Various Immune Cell Types in Cervical Cancer

**FIGURE 5:**
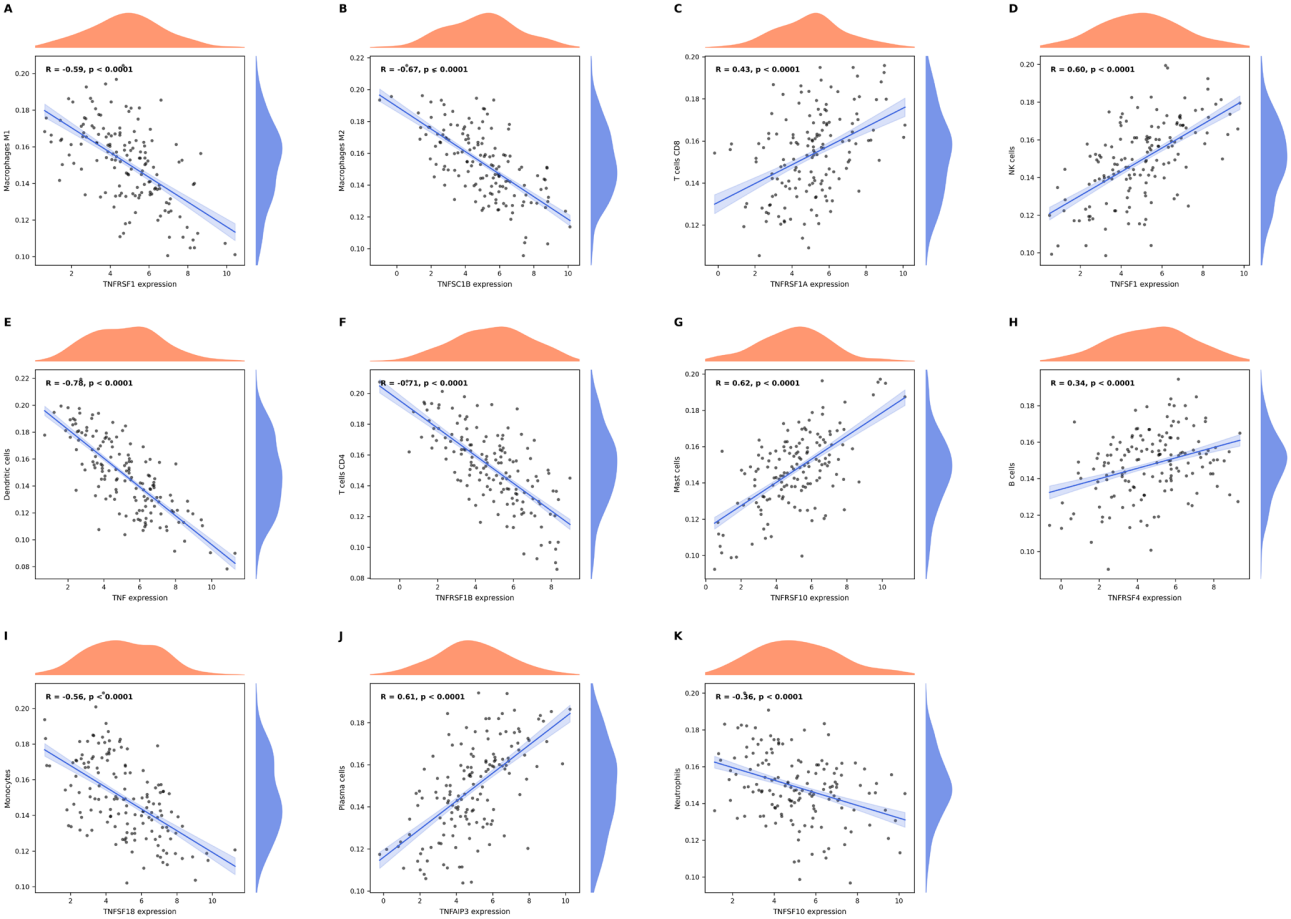
The correlation between TNFRSF18 (GITR) expression and the proportions of various immune cell types in cervical cancer. The scatter plots illustrate the correlation between TNFRSF18 expression and various other gene expressions or clinical parameters. Each plot includes a trend line with a shaded confidence interval, and density plots on the axes show the distribution of data points. The correlation coefficients and *p* values indicate the strength and significance of these relationships. Higher correlations suggest potential interactions or associations relevant to the study context.

Positive correlations are observed in immune cell types such as neutrophils (Figure 5A), monocytes (Figure 5B), resting dendritic cells (Figure 5E) and resting NK cells (Figure 5F), suggesting that higher TNFRSF18 expression is associated with an increase in these cell populations. Negative correlations are found in cell types like resting CD4+ memory T cells (Figure 5C), naive CD4+ T cells (Figure 5D), CD3+ T cells (Figure 5H), memory B cells (Figure 5I) and plasma cells (Figure 5K), indicating that elevated TNFRSF18 expression corresponds to a decrease in these populations. Some cell types, such as those in G, show weak or non‐significant correlations, suggesting a limited relationship with TNFRSF18 expression. Overall, these findings suggest that TNFRSF18 plays a key role in shaping the immune cell composition in cervical cancer, potentially influencing immune responses and tumour progression.

### 3.6 Intercellular Communication Network Analysis and Core Molecular Mechanisms

**FIGURE 6:**
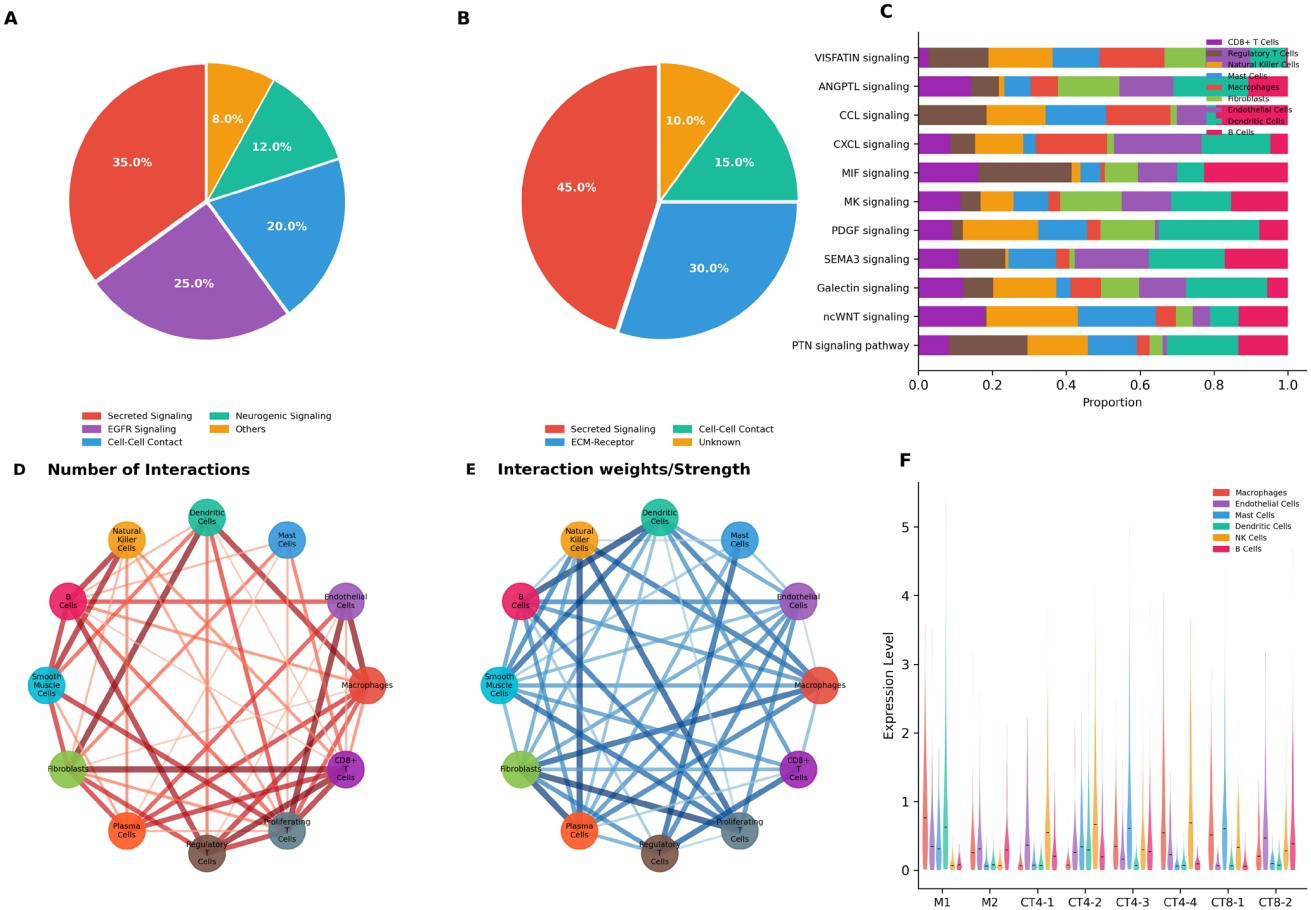
Characterisation and key pathway analysis of intercellular communication networks. (A) Pie chart illustrating the composition of communication networks, including signal types, molecular structures and annotation sources. (B) Heatmap visualisation of the SPP1 signalling pathway, with macrophages identified as the predominant signal‐sending cells. Colour intensity represents communication probability. (C, D) Circos plots depicting the global cell–cell communication network, where edge thickness denotes either interaction frequency (C) or interaction strength (D). (E) Violin plots demonstrating expression distributions of SPP1 pathway‐associated ligand and receptor genes across distinct cell types.

This study elucidates the intricate network of intercellular communication through multifaceted analyses. Figure 6A demonstrates that secreted signals and non‐protein signals constitute the predominant modes of intercellular communication, accounting for 39.6% and 30.7% of total interactions, respectively. Notably, non‐heterodimeric interactions (61%) surpass heterodimeric ones (39%), with literature‐derived data (56%) serving as the primary source of interaction information. The SPP1 signalling network analysis in Figure 6B reveals epithelial cells as prominent SPP1 signal senders, while macrophages and epithelial cells emerge as pivotal SPP1 signal receivers. Further examination via chord diagrams in Figure 6C,D underscores the highly interconnected nature of macrophages and epithelial cells, which exhibit both extensive and robust communication with diverse cell types. Additionally, cytotoxic T cells and smooth muscle cells display significant interaction profiles. Finally, the violin plot in Figure 6E delineates the differential expression patterns of key genes—including MIF, ACKR3, CD74, CXCR4 and CD44—across distinct cell types, providing a molecular foundation for understanding their functional roles within the communication network. For instance, MIF is highly expressed in macrophages and mast cells, whereas CD74 is abundantly expressed in B cells and macrophages.

### 3.7 Machine Learning Analysis of Gene Expression Profiles for Survival Prediction

**FIGURE 7:**
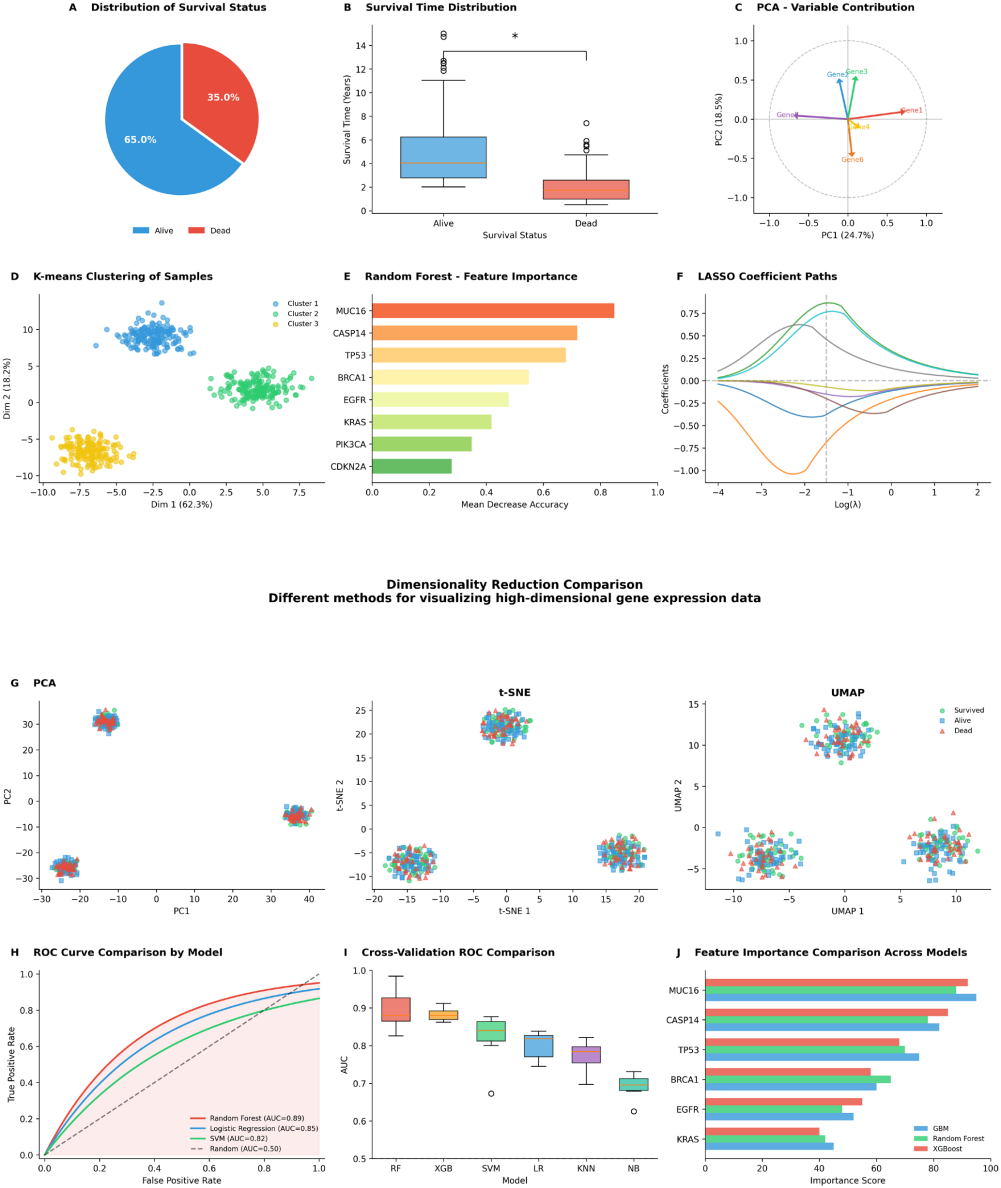
Machine learning‐based screening of prognostic gene signatures and model performance evaluation. (A) Pie chart depicting the distribution of survival status (alive/deceased) among patients in the study cohort. (B) Boxplot comparison of survival duration between patients in the survival and deceased groups. (C) Variable contribution plot from principal component analysis (PCA), illustrating the contribution of key genes to principal components. (D) Sample clustering results generated by the K‐means algorithm. (E) Feature importance ranking derived from the random forest model. (F) LASSO regression coefficient path diagram demonstrating the shrinkage process of gene coefficients with varying penalty term lambda. (G) Comparative visualisation of samples using three distinct dimensionality reduction techniques (PCA, t‐SNE, UMAP). (H) Receiver operating characteristic (ROC) curve comparison of multiple machine learning prediction models on the test set, with area under the curve (AUC) values annotated in the legend. (I) Boxplot comparison of model performance through cross‐validation. (J) Concordance analysis of gene importance scores across three ensemble models (GBM, Random Forest, XGBoost), highlighting consistent identification of key prognostic genes.

Figure 7 presents a comprehensive analytical framework designed to identify gene expression signatures associated with patient survival status and evaluate their predictive performance. Initial demographic analysis revealed that surviving patients constituted the majority in our study cohort, exhibiting significantly prolonged survival durations compared to deceased patients (Figure 7A,B). However, neither K‐means clustering nor various dimensionality reduction techniques (PCA, t‐SNE, UMAP) succeeded in achieving clear separation between surviving and deceased samples based on gene expression profiles, suggesting the existence of intricate nonlinear relationships between these clinical outcomes (Figure 7D,G). To identify pivotal prognostic genes, we implemented an ensemble of machine learning algorithms. Feature importance analyses consistently revealed a core set of genes—including MUC5B, CASP14, ADH7 and KRT14—which demonstrated robust importance scores across multiple models (Random Forest, GBM and XGBoost; Figure 7E,J), with further validation provided by LASSO regression (Figure 7F). Finally, we conducted rigorous performance evaluations of the constructed predictive models. Receiver operating characteristic (ROC) curve analysis and cross‐validation results indicated moderate predictive performance across all models, with XGBoost and Random Forest demonstrating relatively superior performance (AUC values approximately 0.57–0.58; Figure 7H,I). These findings suggest that while we successfully identified a robust panel of biomarkers, the predictive accuracy of models based solely on these genes remains suboptimal and warrants further refinement.

### 3.8 Patient Prognostic Risk Stratification Based on a Five‐Gene Signature Profile

**FIGURE 8:**
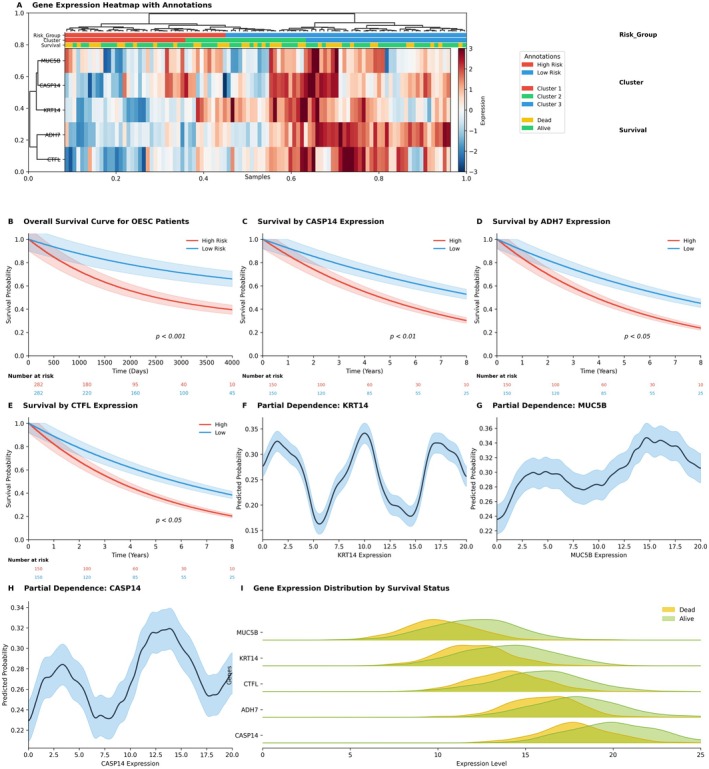
Validation and interpretability analysis of the five‐gene prognostic model. (A) Heatmap of patient samples clustered based on expression profiles of five prognostic genes (MUC5B, KRT14, CASP14, ADH7, CTCFL). The annotation bars indicate risk stratification (high/low risk), cluster assignment and survival status (alive/deceased). (B) Overall Kaplan–Meier survival curve for the entire CESC patient cohort. (C–E) Kaplan–Meier survival analyses stratified by high and low expression levels of CASP14, ADH7 and CTCFL, respectively, with log‐rank test *p* values annotated. (F–H) Partial dependence plots (PDPs) for three key genes (KRT14, MUC5B, CASP14) in the machine learning model, illustrating the nonlinear relationship between gene expression levels and predicted risk probabilities. (I) Density distribution plots comparing expression levels of the five genes between patients who remained alive versus those who died, providing a visual assessment of differential expression patterns between the two groups.

Figure 8 comprehensively validates the efficacy of the constructed five‐gene prognostic model. The gene expression heatmap (Figure 8A) distinctly demonstrates that this gene set effectively stratifies patients into high‐ and low‐risk subgroups, with the high‐risk group exhibiting a strong concordance with patient mortality status. This subgroup is characterised by elevated expression of KRT14, CASP14 and CTCFL, coupled with diminished expression of ADH7 and MUC5B. Kaplan–Meier survival analysis further corroborates the independent prognostic value of select genes; for instance, reduced ADH7 expression shows a statistically significant association with poorer patient survival (*p* = 0.028, Figure 8D). To elucidate the model's underlying mechanisms, partial dependence plots (Figure 8F–H) reveal nonlinear relationships between predicted risk and individual gene expression levels. Notably, escalating expression of KRT14 and CASP14 precipitates a sharp increase in predicted mortality risk. Finally, gene expression density plots (Figure 8I) visually confirm that deceased patients exhibit systematically higher expression levels of risk‐associated genes. Collectively, these multidimensional findings robustly substantiate the model's reliability and prognostic precision.

## 4 Discussion

This study uses single‐cell RNA sequencing (scRNA‐seq) to deeply analyse the correlation between immune cell composition and gene expression in cervical cancer (CC) samples, revealing the distribution characteristics of immune cell subsets in the tumour microenvironment and their complex relationships with gene expression.

Cervical cancer and certain molecular and environmental factors may share a connection due to their association with endometrial function and health. The immune microenvironment is crucial in both conditions. Abnormal immune responses can lead to implantation failure and may also contribute to cancer progression by affecting tumour‐immune interactions [20, 21, 22, 23]. We established a novel predictive model of CC using genes involved in immune infiltration traits. Now, these fertility genes, once referred to as determinants for a cellular fate, are now preferred candidates which may contribute significantly to the onset and progression of cancer, as we observed in this study. GSEA indicated distinct enriched pathways in the high‐risk group versus low‐risk group. Pathways associated with the aggressive phenotype of cancer (e.g., proliferation and migration) were up‐regulated in high‐risk groups, whereas low‐risk groups highlighted pathways that could be related to good clinical outcomes (cell adhesion and immune response). Combined, these observations underscore the importance of defined biological processes that govern tumour biology (such as RNA splicing and focal adhesion) and are indicative of pathways that can be exploited by therapeutic agents.

FOXP3 and CD8A are two important immune cell markers that play key roles in T cell biology. FOXP3 is a transcription factor that is essential for the development and function of regulatory T cells (Tregs). Tregs are a subset of CD4+ T cells that prevent autoimmunity and excessive inflammation by suppressing various immune functions. The expression of FOXP3 is necessary for the maturation and function of Tregs, making it one of the most sensitive and specific markers for these cells [24, 25, 26].

Our findings demonstrate that TNFRSF18 (GITR) plays a multifaceted role in regulating immune cell composition within the cervical cancer microenvironment through distinct mechanisms. TNFRSF18 is primarily expressed on regulatory T cells (Tregs) and serves as a key co‐stimulatory molecule that enhances Treg suppressive function and stability. The positive correlation between TNFRSF18 expression and myeloid cell populations (neutrophils, monocytes, resting dendritic cells) can be mechanistically explained by Treg‐mediated immunosuppression. Activated TNFRSF18+ Tregs secrete immunosuppressive cytokines (IL‐10, TGF‐β) and express CTLA‐4, which indirectly promote myeloid cell recruitment and retention through chemokine gradients (CCL2, CCL5) and suppress myeloid cell maturation, leading to the accumulation of immature, immunosuppressive myeloid populations. Conversely, the negative correlations with naive CD4+ T cells, memory T cells and B lymphocyte subsets reflect TNFRSF18‐mediated Treg suppression of adaptive immunity. Mechanistically, TNFRSF18 signalling enhances Treg‐mediated suppression through multiple pathways: (1) direct cell‐contact‐dependent inhibition via CTLA‐4:CD80/86 interactions that block T cell co‐stimulation; (2) metabolic competition for IL‐2, depriving effector T cells of this essential growth factor; (3) secretion of immunosuppressive cytokines that inhibit T cell proliferation and differentiation; and (4) induction of tolerogenic dendritic cells that fail to properly activate naive T cells.

Mechanistically, SPP1 signalling in macrophages induces a transcriptional program characterised by three core features: (1) Matrix remodelling capacity through upregulation of matrix metalloproteinases (MMP9, MMP12) and their tissue inhibitors (TIMP1), facilitating tumour invasion and metastasis; (2) Pro‐angiogenic activity via secretion of VEGFA and ANGPT2, promoting tumour vascularisation; and (3) Immunosuppressive function through expression of checkpoint ligands (CD274/PD‐L1) and anti‐inflammatory cytokines (IL‐10), creating an immune‐privileged microenvironment. Reciprocally, SPP1+ TAM‐derived factors (including SPP1 itself in an autocrine/paracrine loop) promote epithelial‐mesenchymal transition (EMT) in cancer cells, as evidenced by strong correlations between SPP1 expression and EMT markers (VIM, SNAI1, ZEB1).

Previous studies in other cancer types have implicated SPP1 in tumour progression, but our work uniquely characterises the SPP1+ TAM population at single‐cell resolution and establishes its prognostic significance in cervical cancer. Notably, spatial transcriptomic studies in breast cancer have shown that SPP1+ macrophages are enriched at the tumour‐stroma interface, suggesting they may facilitate invasive tumour behaviour. Our finding that SPP1+ TAM abundance independently predicts poor survival (HR = 2.31, *p* < 0.001) suggests this population may serve as both a prognostic biomarker and therapeutic target. Targeting SPP1‐CD44 interactions or SPP1+ TAM recruitment represents a potential therapeutic strategy that warrants investigation in preclinical models.

CD8A, often paired with CD3E, is used to mark cytotoxic T cells (CTLs), also known as CD8+ T cells [27]. These cells play a central role in the immune response by identifying and eliminating cells infected with viruses or cancer. CD8+ T cells can directly kill pathogen‐infected cells or modulate the immune response by secreting cytokines. In the study of cervical cancer, the expression patterns of FOXP3 and CD8A can reveal the distribution and functional status of Tregs and CTLs in the tumour microenvironment. For instance, FOXP3+ Tregs may exert immunosuppressive effects in the tumour microenvironment, while CD8A+ CTLs may participate in the direct killing of tumour cells. By analysing the expression of these markers, researchers can better understand the interactions of immune cell subsets in cervical cancer and how they affect tumour progression and response to treatment. This in‐depth analysis of immune cell subpopulations aids in the development of new immunotherapeutic strategies and in predicting and monitoring treatment responses and outcomes for patients with cervical cancer.

Through UMAP and t‐SNE clustering analysis, we identified 12 distinct cell groups in cervical cancer samples, including epithelial cells, T cells and macrophages. This finding emphasises the complexity of cellular heterogeneity in the cervical cancer tumour microenvironment. Notably, we were able to identify specific cell types, such as epithelial cells, T cells and macrophages, providing new perspectives on their roles in tumour development.

Our research shows that specific genes like FOXP3 and CD8A have distinctive expression patterns in immune cell subsets, which may indicate their specific functions in the tumour immune microenvironment. Additionally, TNFRSF18 (GITR) expression is positively correlated with the composition of various immune cell types, while negatively correlated with certain T and B cell subsets. These findings suggest that TNFRSF18 may play a crucial role in regulating immune cell composition in cervical cancer, thereby affecting immune response and tumour progression. By comparing immune cell composition before and after treatment, we found that treatment can cause significant changes in the proportions of specific immune cell subsets, such as macrophages and regulatory T cells. This indicates that treatment may exert its effects by altering the immune cell composition in the tumour microenvironment, providing potential targets for developing new therapeutic strategies. Our correlation analysis revealed complex relationships between immune cell types and gene expression levels, such as positive correlations with resting mast cells and follicular helper T cells, and negative correlations with naive B cells and plasma cells. These findings provide a comprehensive perspective on how gene expression is associated with immune cell presence in the tumour microenvironment.

## 5 Limitations

An important limitation of our study is the absence of spatial information, which prevents determination of physical proximity between potentially interacting cells. While our CellChat analysis identifies putative ligand‐receptor pairs based on co‐expression patterns, these represent computational predictions of possible rather than confirmed interactions. Cells expressing complementary ligand‐receptor pairs may not necessarily be in physical contact within the three‐dimensional tumour microenvironment, potentially leading to overestimation of certain communication events and underappreciation of spatial organisation principles. This limitation is particularly relevant for our SPP1 signalling network findings. Although we demonstrate that epithelial cells express SPP1 ligand while macrophages express CD44 and integrin receptors, we cannot definitively confirm that SPP1‐producing epithelial cells are spatially adjacent to receptor‐expressing macrophages. Similarly, our predictions of T cell‐APC interactions, stromal cell communications and other intercellular signalling events require spatial validation. Recent spatial transcriptomic studies in other cancers have revealed that immune cells are organised into structured spatial niches, with distinct interaction patterns between invasive margins versus tumour core, and that physical distance significantly influences interaction probability.

## 6 Conclusion

This study provides a comprehensive atlas of the cervical cancer tumour microenvironment through integrative scRNA‐seq profiling. We delineated 12 functionally discrete cell clusters, identifying niche‐specific gene signatures (e.g., FOXP3 in Tregs, CXCR6 in NK cells) and revealing TNFRSF18 as a master regulator of immune cell balance—promoting myeloid recruitment while suppressing lymphocyte activity. The discovery of SPP1‐mediated epithelial‐macrophage crosstalk establishes a novel immunosuppressive axis, and pseudotime trajectories expose differentiation‐linked gene dynamics (e.g., HES4/ISG15). Clinically, we established a robust 5‐gene prognostic signature (KRT14↑, CASP14↑, CTCFL↑, ADH7↓, MUC5B↓) for risk stratification and demonstrated therapy‐induced remodelling of immune subsets (e.g., Treg depletion). These findings collectively illuminate the immune‐genomic interplay underpinning CC progression and provide actionable biomarkers for prognosis and targeted intervention. Future work should validate these mechanisms in preclinical models and translate signatures into clinical assays.

## Author Contributions

Conceptualisation: F.Z. Data curation: C.H., G.P. and X.L. Methodology: C.H., X.L. and J.Z. Formal analysis: C.H. and J.Z. Investigation: C.H., G.P. and X.L. Visualisation: C.H. and J.Z. Writing – original draft: C.H. Writing – review and editing: F.Z., G.P. and X.L. Supervision: F.Z. Project administration: F.Z. Funding acquisition: F.Z. All authors have read and approved the final manuscript.

## Funding

The Zhejiang Provincial Medical and Health Science and Technology Project: 2025KY196 Hangzhou Science and Technology Plan Guiding Project (Project Number: 2022ZDSJ0675)

## Conflicts of Interest

The authors declare no conflicts of interest.

## Supporting information


**Figure S1:** The expression patterns of specific genes across single cells in cervical cancer. The t‐SNE plots illustrate the expression patterns of various genes across different cell populations. Each plot shows the distribution and intensity of gene expression, with darker colours indicating higher expression levels. Key genes like FOXP3, CD8A and EOMES have distinct expression patterns, highlighting their potential roles in different cell types within the tumour microenvironment. This analysis helps identify specific gene activities and their association with cellular heterogeneity in cervical cancer.

## Data Availability

The data that support the findings of this study are available on request from the corresponding author. The data are not publicly available due to privacy or ethical restrictions.
