# Supplementary material for: Single‐Cell RNA Sequencing Analysis Reveals Correlation Between Immune Cell Composition and Gene Expression in Cervical Cancer
Source: J Cell Mol Med. 2026 Jan 26;30(2):e70998. doi: 10.1111/jcmm.70998 (PMC12835611; doi:10.1111/jcmm.70998)
Supplement: Supplementary file 1 — Figure S1: The expression patterns of specific genes across single cells in cervical cancer. The t‐SNE plots illustrate the expression patterns of various genes across different cell populations. Each plot shows the distribution and intensity of gene expression, with darker colours indicating higher expression levels. Key genes like FOXP3, CD8A and EOMES have distinct expression patterns, highlighting their potential roles in different cell types within the tumour microenvironment. This analysis helps identify specific gene activities and their association with cellular heterogeneity in cervical cancer. [file JCMM-30-e70998-s001.docx]

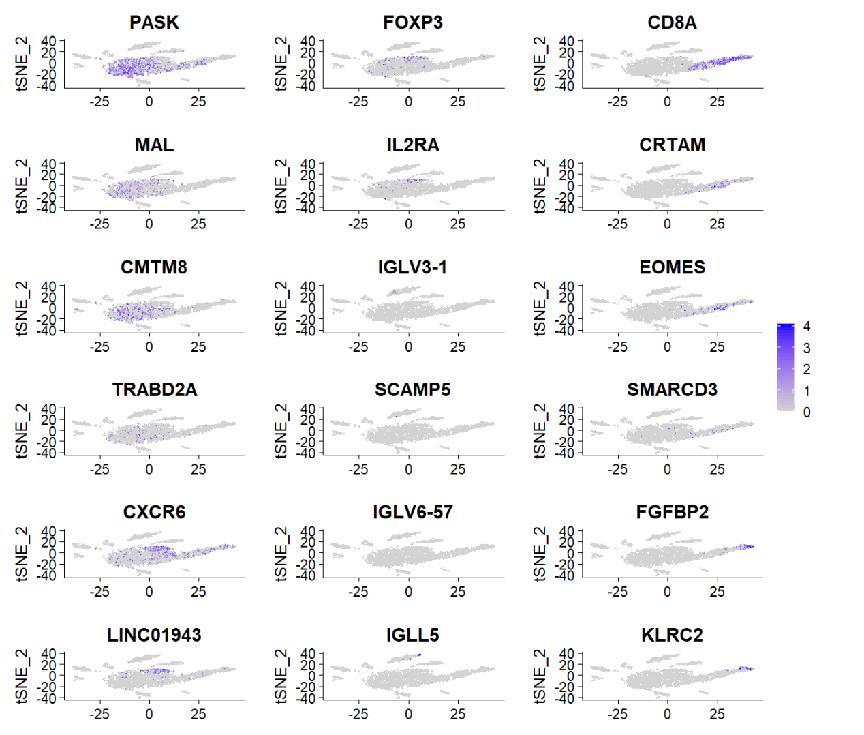


**Supplementary Figure 1. The expression patterns of specific genes across single cells in cervical cancer.** The t-SNE plots illustrate the expression patterns of various genes across different cell populations. Each plot shows the distribution and intensity of gene expression, with darker colors indicating higher expression levels. Key genes like FOXP3, CD8A, and EOMES have distinct expression patterns, highlighting their potential roles in different cell types within the tumor microenvironment. This analysis helps identify specific gene activities and their association with cellular heterogeneity in cervical cancer.
